# *‘I was in between wars’* – Perspectives of mothers about consequences of HIV exposure for their uninfected children during early childhood – Qualitative insights from Worcester, South Africa

**DOI:** 10.1080/15381501.2025.2582087

**Published:** 2025-11-10

**Authors:** Himani Pandya, Xanthe Hunt, Peter S. Nyasulu, Amy L. Slogrove

**Affiliations:** a Department of Global Health, Faculty of Medicine & Health Sciences, Stellenbosch University, Western Cape, South Africa; b Africa Health Research Institute, Somkhele, KwaZulu Natal, South Africa; c Institute for Life Course Health Research, Department of Global Health, Stellenbosch University, South Africa; d Mental health, Alcohol, Substance Use and Tobacco Research Unit (MAST-RU), South African Medical Research Council, Western Cape, South Africa; e Division of Epidemiology & Biostatistics, Department of Global Health, Faculty of Medicine & Health Sciences, Stellenbosch University, Western Cape, South Africa; f School of Public Health, Faculty of Health Sciences, University of the Witwatersrand, Johannesburg, South Africa; g Department of Pediatrics and Child Health, Faculty of Medicine & Health Sciences, Stellenbosch University, Western Cape, South Africa

**Keywords:** HIV, VHT, HEU, early childhood development, caregivers’ concerns, motherhood, and HIV

## Abstract

This qualitative study explores concerns of mothers living with HIV about the consequences of HIV exposure for their children during pregnancy and early childhood, before and beyond a final HIV negative diagnosis. Conducted in South Africa’s Western Cape between January and April 2024, the study involved semi-structured interviews with 20 mothers whose children HIV-exposed, over one year old, tested HIV-negative at 12 months. The study revealed that maternal fear of vertical HIV transmission persisted throughout pregnancy and early childhood. It was triggered by breastfeeding decisions, repeated HIV testing, sickness or growth faltering in the child, and doubts about antiretroviral effectiveness. Mothers adopted coping strategies such as strict medication adherence, routine clinic visits, and heightened hygiene practices. The study indicates that comprehensive, ongoing support for mothers living with HIV throughout the early years of motherhood, including breastfeeding guidance, clear information on HIV testing protocols, and guidance on long-term implications of HIV exposure can alleviate maternal stress.

## Introduction

Mothers play a significant role in the caregiving and nurturing of all children, including those affected by HIV. While the universal availability of Antiretroviral Therapy (ART) has improved overall health and longevity of mothers living with HIV, going through pregnancy and the early years of motherhood in the context of living with HIV can add additional stressors and demands to women’s daily life. Mothers with HIV must play the dual role of a patient as well as a caregiver, navigating a long journey dealing with their own ART, delivery experiences, breastfeeding dilemmas, fear of HIV transmission, HIV testing and ART prophylaxis for their young children exposed to HIV (Fauk et al., 2022; Jolle et al., 2022; Nguyen et al., 2024; Tuthill et al., 2021; Velapi et al., 2023). A range of challenges, including those related to mothers’ own health (mental or physical), socio-economic status and family environment could further complicate their caregiving journey (Ashaba et al., 2017; Silveira et al., 2016).

Evidence shows that poor mental health among mothers living with HIV can adversely affect their caregiving practices and efficiency, parent–child interaction, early childhood behavior and cognitive development, and contributes to the sub-optimal health outcomes observed in some children who are exposed to HIV (Chi et al., 2015; Rochat et al., 2017; Schulte et al., 2019; Silima et al., 2024; Steventon Roberts et al., 2022). In this regard, it is important to understand what mothers living with HIV experience, mentally and emotionally, during the period of their pregnancy and after birth. Better understanding of these experiences – including their feelings and concerns regarding HIV and its consequences for their children – can inform supportive interventions (Ashaba et al., 2017).

Current literature on caregivers’ concerns about children in the context of HIV mostly centers on children who are confirmed to be living with HIV (Atanuriba et al., 2021; Joyce et al., 2022; Moshoeshoe & Madiba, 2021). The existing studies, which are few in number, are mostly quantitative, do not focus on early childhood, and explore mothers’ concerns mostly in terms of their fears about modes of HIV transmission, stigma/discrimination, and HIV status disclosure (Ashaba et al., 2017; Hayati et al., 2023; Joyce et al., 2022; Moshoeshoe & Madiba, 2021; Nordberg et al., 2020; Shannon & Lee, 2008; Silveira et al., 2016; Worku et al., 2023). However, not much is known about the concerns of mothers living with HIV for their children after vertical HIV transmission has been excluded through age-appropriate testing. We also do not know much about how these mothers’ concerns and feelings evolve throughout their caregiving journey from pregnancy through their child’s early years, what factors affect their state of mind (trigger or alleviate these concerns) and how these concerns affect their caregiving practices, particularly from a qualitative perspective.

This study seeks to explore mothers’ perceptions, feelings, and concerns regarding the effects of HIV and its consequences for their children who are HIV exposed and uninfected (HEU) (before and beyond a final HIV negative diagnosis), identify factors which influence these concerns and examine how they shape mothers’ caregiving practices. We believe that a nuanced understanding of these concerns can better support mothers in dealing with their stress and improve their caregiving journey during the critical early years of their child’s life.

## Methods

This was a qualitative, descriptive study nested within a larger prospective longitudinal cohort study called CHERISH (Children HIV Exposed Uninfected - Research to Inform Survival and Health) conducted in the Western Cape (WC) province of South Africa (SA) (Slogrove et al., 2023).

The Western Cape is one of nine provinces in SA with a population of 6.8 million. The WC Government Department of Health and Wellness serves the healthcare needs of 75%-85% of the population including ±110,000 pregnant women with ±90,000 deliveries annually. The WC antenatal HIV prevalence is 16.3% with 72.8% of all pregnant women living with HIV (who knew their status) on ART prior to pregnancy in 2022. (Kufa-Chakezha et al., 2024) Vertical HIV transmission in the province is 2% at 14weeks of age (Anderson et al., 2024).

As part of CHERISH, multiple cohort sites were established in WC. For the purpose of this study, mothers were sampled from the CHERISH prospective cohort based in Breede Valley which is a rural community around the town of Worcester in WC. Antenatal care is provided to ±3,600 women annually, with an antenatal HIV prevalence of 16%. Primary level antenatal care is provided at Worcester Midwife Obstetric Unit (MOU) and six smaller primary health care clinics that are supported by Worcester Provincial Hospital for secondary, and Tygerberg Hospital in Cape Town for tertiary care.

All women enrolled in the CHERISH prospective cohort who had a child HEU (without in-utero or intra-partum transmission of HIV confirmed through HIV-DNA-PCR (polymerase chain reaction) at birth and 10weeks and negative HIV rapid antibody test at 12 months of age) above one year of age by end of 2023 were eligible for participation in this study. Mothers who met the above criteria were sequentially approached and sampled for this study through convenience sampling. Additionally, children who are HEU from potential high-risk groups (known from literature including low birth weight, small for gestational age, increased episodes of illness or hospitalizations, and developmental delays) were purposively sampled to capture any differences in mothers’ concerns. (based on data available from CHERISH database)

Data collection was done between January and April 2024. Mothers were approached during their scheduled CHERISH study visit at the Worcester Campus Clinical Research site for CHERISH. The objectives and interview process for this study were explained to the mothers and written informed consent was obtained, from those who agreed to participate. All participants were offered a small remuneration for time, inconvenience, and expense.

Twenty semi-structured, in-depth interviews were conducted, each lasting approximately 1-1.5 hours. Based on the mothers’ preferred languages, three interviews were in English, conducted by the first author/primary investigator (PI), while 17 were in isiXhosa, conducted by a bilingual research assistant (RA) under PI’s supervision. The RA received training on in-depth interviewing, and regular debrief sessions were held to address question wording, cultural relevance, building trust, managing sensitive topics, and dealing with any other challenges encountered during interviews. Interviews were guided by open-ended questions, linked to the study objectives and information gained from a scoping review which preceded this study (Pandya et al., 2025). The interview guide was translated to isiXhosa and piloted on a small sample of mothers, results of which were used for subsequent revisions. (Appendix 1)

Data from in-depth interviews were transcribed verbatim and translated to English (for isiXhosa interviews) by trained bilingual transcribers. Quality control was done by a trained member of the transcription team, who reviewed the audios and transcripts to ensure consistency and quality. All transcripts were analyzed using MaxQDA software (VerBi Software MAXQDA 2022, 2021).

Data was analyzed according to the six steps of reflexive thematic analysis suggested by *Braun and Clarke* which is an interpretive and reflexive approach to identify and analyze patterns of meanings within qualitative data (Braun et al., 2023; Braun & Clarke, 2019). We chose this approach to engage with perceptions of mothers in depth and nuance and interpret their meanings in a reflexive manner specifically to answer questions such as ‘how mothers make sense of HIV exposure for their child’ and ‘what factors shape their concerns’. Firstly, all transcripts were reviewed repeatedly to get familiar with the data and develop an intuitive understanding. This was followed by systematic identification of initial interpretive codes in the form of meaningful text segments which relate to the research question. Text segments from the transcripts were coded using predominantly inductive codes (emerging from the transcripts) as well as deductive codes (identified though literature review). A codebook was developed with detailed definitions of each code to ensure that they are consistently applied to all transcripts. Ongoing analysis and familiarity with data allowed further revision and evolution of codes. A second coder (one of the co-authors) coded a few transcripts using the codes and definitions that were developed by the first coder (PI). Codes were then interpreted and grouped to develop and refine coherent and meaningful themes and sub-themes (emerging issues and concepts). Finally, all themes were integrated to a cohesive narrative (supported by examples and quotes from participants) which has been presented in the results. Data analysis was initiated after conducting the first few interviews and concurrent analysis allowed for revision of questions to improve the richness and quality of information obtained.

Ethical Clearance was obtained from the Health Research Ethics Committee (HREC) at Stellenbosch University. (approval number - S22/09/182) As part of risk management protocol, mothers were informed about their right to pause or stop the interview at any point. Given the sensitive nature of this topic, we ensured that at any point during the interview, if a mother was distressed, the researcher paused the session and offered emotional assurance. If it was found that a particular mother or her child/children needed additional care and support related to health or other needs, they were referred to the closest available, appropriate service provider/s for ancillary care (e.g. social worker/psychologic counsellor/community health worker/clinic or hospital).

## Results

Our sample consisted of 20 mothers raising a child vertically exposed to HIV and uninfected. (all with negative birth and 10-week HIV-DNA-PCR test and tested negative at 12 months with a rapid antibody test) Mothers’ ages ranged from 22 to 41 years with a mean of 33 years. Most of them had junior high school education (up to grade 9), few had senior high school education (grade 10 to 12) and one of them did not go to a school. In terms of employment, except 4 mothers employed full time and 1 mother pursuing college education, majority of the mothers (75%) were unemployed at the time of data collection since they were temporary workers in grape farms and lost their jobs when the harvest season was over (3 to 4 months in a year). More than half of the mothers (55%) were not cohabitating with their partners. Mothers cared for one to five children, with an age range from 1 month (age of youngest child) to 22 years (age of oldest child). Out of these children, some were HIV unexposed, and others were HEU. The age of enrolled children ranged from 12.05 to 24 months with a mean of 16.7 months. None of the mothers were caring for a child living with HIV. For this study, mothers were asked about their concerns for their child HEU enrolled in the CHERISH cohort. Socio-demographic characteristics of mothers and their children are shown in Table 1.

The following four themes emerged from our data analysis:

1. Early years of mothering – uncertainty, stress, and anxiety
2. Factors driving mothers’ uncertainty, stress, and anxiety about their children who are HIV exposed and uninfected
3. Effect of uncertainty, stress, and anxiety on mothers’ caregiving practices toward children who are HIV exposed and uninfected
4. Mothers’ understanding about consequences of HIV exposure for their children who are HIV exposed and uninfected

### Early years of mothering – uncertainty, stress, and anxiety

**Figure 1.**
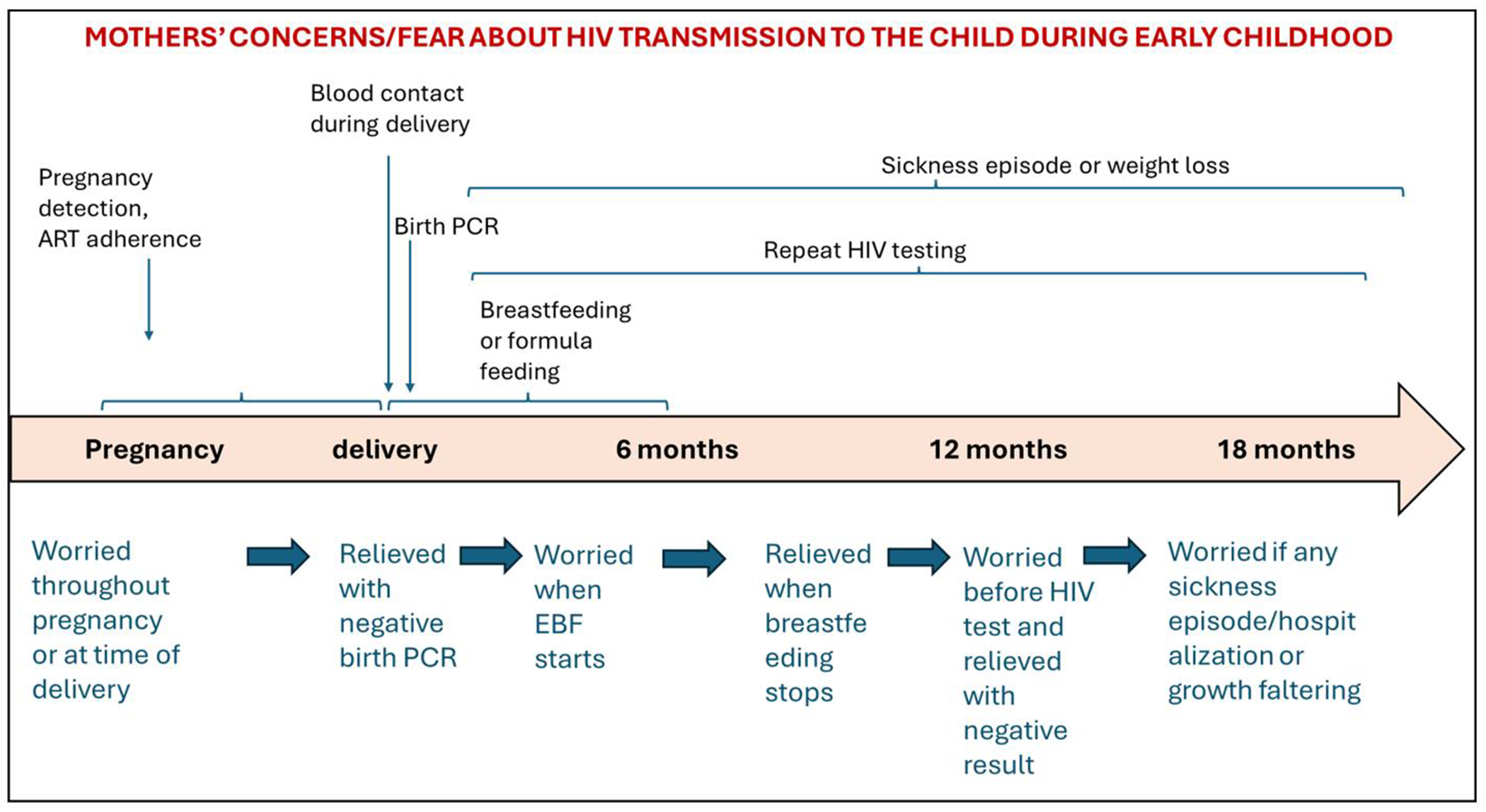
Mothers’ concerns regarding HIV for their children HIV exposed and uninfected – An evolving journey through pregnancy and early childhood.

Mothers’ narratives indicate that during pregnancy and most part of early childhood, they remained in a state of fear and uncertainty regarding potential transmission of HIV to their child and its implications. Further exploration revealed that their fear fluctuated throughout the early years of motherhood, according to distinct stages of their caregiving journey. (Figure 1)

For majority of mothers, this fear *started during pregnancy* particularly among those with first time HIV (7/20) as they expressed concerns about potential HIV transmission to their unborn child. When diagnosed as pregnant and with HIV, many mothers questioned the possibility of delivering a child who is HIV-free, given that the baby develops in the mother’s body containing HIV. These mothers not only wanted to protect their unborn children from HIV so that they could grow up like other children (unexposed to HIV) but also desired to maintain their own health and longevity, which could enhance their caregiving capacity after birth. At this stage, guidance and support from the clinic staff significantly alleviated their stress and motivated adherence to ART. Reassured by health workers, many of these mothers felt convinced and relieved of their fear for the rest of their pregnancy.

If something is inside me and I am sick, how possible it is for my baby inside my body to be negative (HIV). Because my baby is going to have the same blood. I am doing everything that the doctor has told me, but I still did not believe that the baby can be negative. (M9)

A mother, who had an unplanned pregnancy at 42 years and was newly diagnosed with HIV, shared her guilt and concerns below:

It stressed me a lot when I was pregnant, what will I do with this child at 42, with HIV … [But] nurses explained to me that no, when you are pregnant, it does not mean you are extremely sick. That is when my mind came back. I ended up forgiving myself. now I am pregnant with my baby, I will get him in a right way. (M18)

However, for some mothers, this fear *persisted throughout pregnancy until delivery*. One mother even wondered about her child’s contact with her blood during the delivery process.

They used to say when the baby comes, you are opened, he has blood … What they do if the child contacts your blood? I was thinking for real, if she comes out to be HIV positive, what will I do? But if I am taking my treatment correctly, how can that happen. There were many things on my mind. (M12)

For mothers, whose doubts persisted throughout pregnancy until birth, the fear of potential HIV transmission was alleviated when the child tested negative at birth. At this stage, they felt relieved that there is no more risk of HIV or its effects to the child. However, this fear reemerged soon when it was time to *decide about breastfeeding*. Several mothers expressed concerns about potential transmission of HIV to the exposed child through their breast milk. (elaborated in upcoming themes)

The first test that they do in the hospital, it encouraged me that I can breastfeed, because this baby is coming from inside of my body but is still negative, and the nurse also explained … .that if you take your medication every day, even the milk that is coming from your breast will not transmit the virus to your baby … .but … .when I came from the hospital, it was not easy, they tested the baby negative, but now I have to put him to my breast! (M9)

Discontinuation of breastfeeding brought relief for many mothers who had previously been concerned. However, for most of these mothers including those who chose not to breastfeed, this concern was triggered each time their *child was tested for HIV*, which occurred multiple times during infancy and beyond. Furthermore, while multiple HIV-negative test results brought relief for mothers, *any episode/s of sickness, hospitalization, or growth faltering during early childhood*, even beyond a final negative test, reactivated their fear as they remained concerned that such episodes could be linked to HIV. (elaborated in upcoming themes)

Among all the mothers interviewed, only three mothers reported being entirely stress free and convinced that their child is completely free from HIV (regardless of the child’s age and stage of caregiving). They believed that as their child was born without HIV, they cannot acquire HIV at a later stage.

### Factors driving mothers’ uncertainty, stress, and anxiety about their children, HIV exposed and uninfected

**Figure 2.**
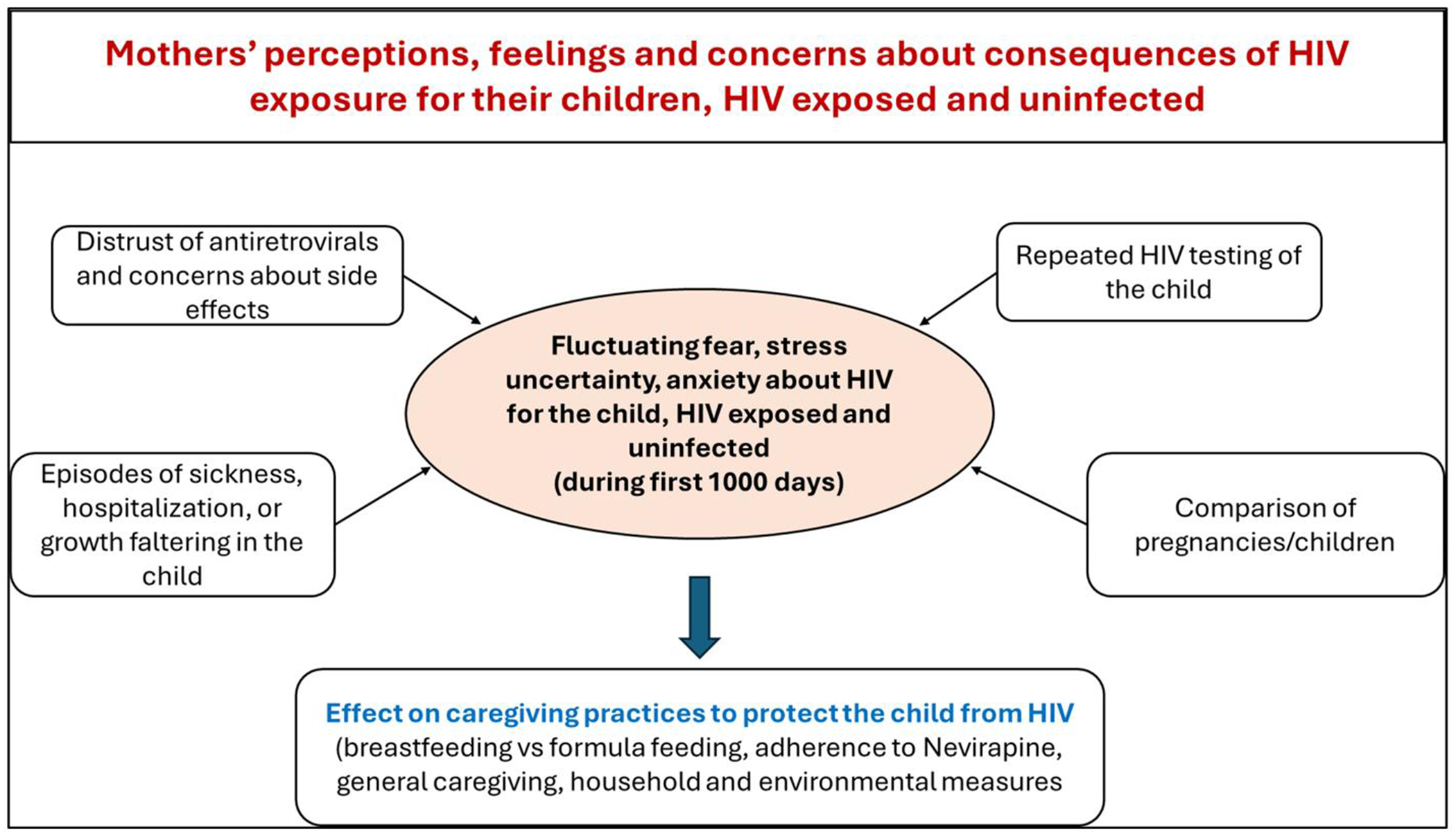
Mothers’ perceptions, feelings, and concerns about consequences of HIV for their children, HIV exposed and uninfected during early childhood.

Mothers’ perceptions, feelings and concerns about HIV transmission were shaped by various inter-related factors, many of which co-existed and influenced each other (Figure 2). While some of these factors alleviated their concerns, others exacerbated them. These factors include the following:

#### Distrust of ART and concerns about side effects

Mothers’ concerns regarding possible HIV transmission to their child were linked to their perceptions about ART. Based on advice received from health workers, majority of mothers expressed trust and confidence in the ability of ART to prevent HIV transmission and reported high adherence. Moreover, mothers who had experienced a previous healthy pregnancy with HIV were more likely to trust ART. However, some mothers doubted the effectiveness of ART.

I just took the medicine (ART), but I was like, is it really going to work? I was thinking that it only helps me fight my HIV but to prevent the transfer to my baby, I do not believe that. When you are stressed, you think a lot of things. (M9)

For some mothers, the fear of HIV transmission was found to be linked with non-adherence to ART. Even though most of them reported good adherence, further probing revealed that some of them had unpleasant experiences with ART, leading to occasional missed doses both during pregnancy and breastfeeding. Factors contributing to non-adherence included stress arising from financial struggles, caregiving burden and strained relation with partner or family. Additionally, side effects such as weakness, fatigue, vomiting, weight loss, headache and dizziness were reported. Other challenges included frequency of doses, unpleasant taste and long or irregular working hours which disrupted the consistency in time of taking ART.

Sometimes I did not drink pills (ART), so my worry was that I wish this child does not get infected (with HIV). These pills annoy me, and I will not take them for a month. They make me nauseous. what irritated me, I drank 2 pills … .in the morning and in the night. So, the problem was I did not keep time. (M4)

In addition to concerns regarding effectiveness, some mothers expressed apprehensions about side effects of ART both for themselves and their child. These included skin pigmentation in mothers and red eyes and rashes in the baby. Moreover, while one mother believed that ART caused her fetus to be big and resulted in cesarean section, another mother wondered if her child’s low weight and small size at birth could be because of ART side-effects. These findings suggest that in the absence of accurate information from health workers, mothers continued to speculate, leading to increased anxiety and reinforcement of misconceptions regarding effects of ART.

These people (fellow patients) will tell you … because you are drinking medication (ART), when pregnant., the baby is going to have rashes and red eyes … … you will have skin pigment. I ask them, when he (baby) is having these rashes, so how is he going to be negative (surprised)? When I asked the nurse, she said, she knew nothing about it. (M9)

#### Repeated HIV testing of the child

Repeated HIV testing of the child triggered fear of HIV transmission for many mothers. Despite being relieved after cessation of breastfeeding, mothers’ concerns would reemerge with each test. These tests were prescribed either as part of routine testing (conducted at birth, 10 weeks, 6 months, 12 months - as part of CHERISH cohort study, 18 months and 6 weeks after cessation of breastfeeding) or during an illness episode. Having to revisit the possibility of HIV in their child over an extended time period and the fear of a positive diagnosis caused ongoing stress and took an emotional toll on these mothers with each test serving as a reminder of their child’s vulnerability.

While some mothers were reassured after multiple HIV negative tests, a few of them were confused and anxious about reasons for repeated testing. They questioned how a child who was born without HIV, could acquire the virus over time and after multiple negative tests, despite mother’s adherence to ART with a good CD4 count and taking necessary precautions. Although mothers were relieved after each negative test, waiting for the results and anticipation of the next test exacerbated their concerns and anxiety.

What confuses and stresses me a lot is when he (child) keeps on getting tested. Because when he was born, he was declared negative. Is it not that if he is drinking his medicine (Nevirapine) while breastfeeding and I am taking my treatment correctly, so why would he be tested again and again? I felt that something (HIV) was found in him. What amazes me is that where would he get the HIV from as he came out without it. I was not coping well. (M12)

Furthermore, despite the negative test at 12 months (designated as the cut off for confirming HEU status in this study), most mothers remained uncertain about the final HIV status of their children. Some mothers felt reassured that the negative test obtained after cessation of breastfeeding was conclusive whereas few others placed their confidence in two to three multiple negative tests done as part of routine testing. Thus, in a mother’s mind, it was not clear as to when her child can be definitively declared HIV negative for life.

For mothers who decided to breastfeed despite their inherent fear of HIV, repeated testing of the child compelled some of them to question their feeding choices and reconsider their decision.

I believed that they (twins) may not have it (HIV), but I had that fear that they may end up getting it. Because that is when I decided that no … to get out of these minds I am in …. I would better stop them from breastfeeding (M5)

Repeated HIV testing also aggravated some mothers’ doubts about ART effectiveness. Mothers questioned the source of possible HIV infection in the child at a later stage, thus reflecting a complete lack of understanding about modes of HIV transmission after birth which resulted in anxiety and confusion.

I took the medication (ART) during pregnancy, but I do not trust it for real. what makes me not to say my child is safe from the virus is that of being tested often … .they test him … .then say its negative … .again get tested … … it is where I get confused. (M10)

Repeated HIV testing also compelled two mothers to question their caregiving practices and precautions taken to protect their child from HIV.

Let us say I was cooking, and I get a cut on my hand, I must stop immediately so that the child cannot spot that food because if she eats that food, she is going to get HIV. But what is confusing me is if she never got in touch with my blood, how can she get infected … … as they keep on testing… .testing… .and testing. I don’t have an answer. (M10)

When probed further, it was revealed that mothers’ stress and anxiety were driven by a lack of understanding about the number of tests prescribed post birth, rationale behind repeated testing and when they could expect definitive confirmation about their child’s HIV negative status.

I asked her (nurse), now the baby is negative, when can I get relieved and be sure. She said wait at least until 6 months for the confirmation of HIV. But I don’t know until what age will he be tested. I believe that three tests have come negative. So, its fine. I don’t know when they will test him again. In the clinic if you ask, they don’t tell you anything. (M9)

On the other hand, some mothers were happy with repeated testing as they wanted to be completely sure that the child is HIV negative. Two mothers insisted on repeated testing since they wanted to be certain so that they could continue breastfeeding for long.

I always wanted them to test my babies every month so that I can put my mind at ease… After 6 months, when I stopped breastfeeding and they did the last test and it was negative, I was like thank God, I am finally done with breastfeeding, and I am done with worries about their HIV status. (M5)

#### Episodes of sickness, hospitalization, or growth faltering in the child

Irrespective of the age of most recent HIV negative test, if their child was growing well and healthy, mothers were less likely to worry about HIV. However, uncertainty and fear resurfaced for some mothers when their child’s health, growth or development differed from siblings or other children (HIV exposed or unexposed), especially after episodes of illness, hospitalization, or growth faltering. They questioned the potential underlying cause, particularly, since they believed that they were providing optimal care and nutrition to the child. Regarding the cause, while some mothers felt confused, others suspected that their child’s condition could be a result of ‘*HIV coming back*’ or ‘*HIV remaining in the child’s blood*.’

One mother was worried that her 18 months old child had three hospitalizations (meningitis, breathing problems), multiple episodes of sickness and weight loss. She expressed:

When this one gets sick, I do not feel fine, I am losing hope. He (child) started getting sick at 3 weeks of age, for us to go and sleep at the hospital. I thought maybe they (health workers) would say it has something to do with HIV or the treatment (ART), but they didn’t say anything. They drew blood on my child, came back with the results and tell me, no it (HIV) is not what is causing this sickness. (M4)

#### Comparison of pregnancies/children

Mothers with previous healthy pregnancies with HIV or healthy older child/ren (HIV exposed) were less worried about HIV transmission as compared to those who had their first pregnancy with HIV. These mothers had more confidence in ART, better knowledge about modes of HIV transmission and what to expect in terms of their child’s growth and health.

I trust the medication (ART) because the first time, I was HIV positive, I was eating my treatment, I didn’t get sick.so I put all of that together with this child’s (second) experience and thought….this works! (M14)

One the other hand, few mothers compared their recent pregnancy (HIV exposed) with their previous pregnancies (HIV unexposed). Those who witnessed any differences in health, growth or development patterns in the exposed child were more likely to be stressed. While some of them were confused about what could cause this difference, others attributed this to HIV or ART exposure during pregnancy.

My first pregnancy, I didn’t experience things that I don’t understand as I was (HIV) negative. Now, I am HIV positive… So, it was difficult and new for me. I will be judging myself that this thing is happening because of HIV. Not up unless I go to the clinic, and they explain to me. but if I don’t have answers, I will be like …. with my second baby, I was drinking medication (ART)….that’s why the baby is like this. I was stressed. I was always thinking, thinking, thinking… …maybe I affected my baby during the pregnancy because of thinking and medication. (M9)

### Effect of uncertainty, stress, and anxiety on mothers’ caregiving practices toward their children who are HIV exposed and uninfected

Mothers’ concerns and fear of HIV for their children affected their caregiving practices and experiences as most of them took extra care and precautions both during pregnancy as well as after birth to alleviate this fear. During pregnancy, in addition to adhering well to ART, mothers paid attention to their own health, ate well, exercised, refrained from smoking and alcohol, adopted safe sexual practices, booked early at the clinic, and attended all antenatal care visits.

Mothers even adopted certain practices and precautions during early childhood which include:

#### Health care related practices

Following guidance from health workers, all mothers adhered to prescribed antiretroviral prophylaxis for their child and ensured that they did not miss any dose. They believed that the ‘*white medicine*’ (Nevirapine) would effectively protect their child from HIV during the breastfeeding period (particularly those who occasionally defaulted from their own ART). Additionally, they also ensured that they attend all the clinic visits for vaccines and adhere to HIV testing schedule. Two mothers diagnosed with tuberculosis (TB) during breastfeeding period followed nurse’s advice to get their child tested for TB and initiated TB prophylaxis.

I protect my child with everything, I once found out that I have TB and then they were asking me who am I staying with at home, will also have to come to be checked. So, my younger child, dr checked her and, she was given pills to prevent TB so that she does not contract it since I have it. (M7)

#### Feeding-related practices

Fear of HIV transmission significantly added to mothers’ concerns and influenced their feeding practices and experiences not only by shaping their feeding choices at birth (whether to initiate breastmilk or formula milk) but also by affecting their motivation to continue breastfeeding later.

I was in between wars. I thought, no man, the nurses would never fool me and say give the baby breast milk she is not going to get infected. I had that little doubt, the child is being given medication so I don’t think she could get HIV. but there is something in my heart, saying hey I wonder what if my child has it (HIV). (M7)

Even though many mothers initiated exclusive breastfeeding, they had underlying doubts whether their breast milk contained HIV and remained in a constant state of anxiety throughout the duration of breastfeeding. Out of these concerns, two mothers decided not to breastfeed at all, whereas others initiated exclusive breastfeeding after a negative birth PCR along with reassurance from health workers that Nevirapine will protect their child from HIV during the breastfeeding period. However, these mothers discontinued before the recommended period of 6 months (between 1 and 4 months). They decided to do so either independently or following an episode of weight loss in the child or breast issues (cracked/small nipples), having to join work or even missing doses of antiretrovirals occasionally. Few mothers stopped breastfeeding prematurely due to the need to resume their job, fearing that other family members might offer water or other liquids to the child (in the mother’s absence).

The time I got pregnant, I told myself I am not gonna breastfeed at all because I am not gonna risk. I breastfed him for almost 4 months exclusively. And then I stopped breastfeeding all together. It’s because the time I was going to work, sometimes, I would forget to eat the pill. So, I said, let me just buy the formula. (M16)

After discontinuation of breastfeeding, most mothers switched to formula feeding either independently or following guidance from dieticians. Moreover, they did not attempt to resume breastfeeding, even after their feeding issues were resolved. However, two mothers could not afford formula milk and continued to breastfeed, driven by persistent doubts about HIV transmission.

#### Household and environmental measures

Some mothers also implemented various measures to protect their household environment to minimize the risk of HIV infection in their child, thus reflecting their concerns about potential sources of HIV transmission to the child, other than breastfeeding. These measures included ensuring the child received proper nutrition, taking precautions to keep the child away from encountering their own blood, refraining from offering food prepared while they had cuts/wounds and keeping their menstrual pads away from the child. Additionally, they avoided leaving the child with others, maintained cleanliness at home, minimized child’s exposure to dirt outside the house, kept the child indoors and regularly sterilized household objects. One mother decided against sending her child to the creche (daycare center for young children) while another took extra care to sanitize the child’s hands regularly.

I am sure there is nothing he is going to get from my side now unless he gets it from outside. You know children play together. Maybe he plays with one that has it (HIV) and maybe he touches this one with a cut, or they touch a used condom whilst the person who used it was sick. Children like putting things in their mouth and eat. I will not know when he has eaten something or touched someone who is sick. My worry is that he could be infected. (M6)

### Mothers’ understanding about consequences of HIV exposure for their children, HIV exposed and uninfected (beyond a negative test)

When asked about their understanding and concerns about ‘*effects*’ or ‘*consequences*’ of HIV exposure for their children beyond a negative test, nearly all mothers perceived this ‘*effect*’ or ‘*consequence*’ as equating to ‘*being infected by HIV*’ or ‘*HIV coming back*’ after birth or after multiple negative tests. For these mothers, being ‘*affected by HIV*’ was synonymous with being ‘*HIV positive*.’ Once the child was born HIV negative, mothers’ foremost concern and priority was that their child should stay negative beyond a birth PCR. None of the mothers were aware of any medium-to-long-term effects of in-utero exposure to HIV or ART, beyond a negative status, during early childhood or later in life. In their view, once the child is tested negative in multiple tests, s/he must stay free from ‘*effects*’ or ‘*consequences*’ of HIV.

However, one of the mothers compared her two pregnancy experiences (with and without HIV) and mentioned that beyond a final negative test, if she experiences anything different in her child (HIV exposed) in future such as poor health or school performance, she would consider it as an effect of ART taken during pregnancy.

Maybe when you are HIV positive, things would make sense after some time. For now, I feel happy that my baby is negative…. Up unless I see that he is delayed like with walking or maybe at school, if the teachers tell me he is not coping, doesn’t work like other kids, or there is something strange about his behavior, then I will start stressing like what is going on but for now, he is growing, and do things that other kids are doing. Like he must be normal. (M9)

## Discussion

To the best of our knowledge, this is the first qualitative study which explores concerns of mothers living with HIV for their children who are HIV exposed and uninfected, during the period of early childhood. The main finding of our study is that mothers remained in a *constant, fluctuating state of stress, anxiety, and uncertainty due to the fear of HIV transmission* to their children HIV exposed, throughout their journey of early motherhood. Their overall experience of pregnancy and early child rearing was significantly affected by constant thinking and worrying as reflected in the words of one of the mothers – ‘*I was always thinking, thinking, thinking, maybe I affected my baby during the pregnancy because of thinking and medication*.’

Past studies have thrown light on mothers’ concerns about vertical HIV transmission, infant’s health and future, which worry them especially during pregnancy, perinatal and post-partum period (Ashaba et al., 2017; Shannon, 2015; Shannon et al., 2008; Shannon & Lee, 2008). This study reinforces existing evidence by demonstrating that mothers’ concerns about HIV start from the time they are confirmed pregnant with HIV and not only continue through delivery and post-partum period, but extend much beyond, throughout early childhood, thus spanning almost across the first 1000 days of motherhood. As part of current vertical HIV transmission prevention programs, most mothers and their children who are HEU are treated similar to children unexposed, after a final HIV negative test around 18 months or 6 weeks post cessation of breastfeeding, whichever comes later) (Slogrove & Cotton, 2016; Sugandhi et al., 2013). Findings from this study highlight the need to consider the duration of mothers’ concerns and support them throughout the early years of motherhood to improve their caregiving experiences. Instead of once off counseling, mothers need to be handheld throughout this journey depending on how their concerns and state of mind evolves.

Our findings also demonstrate that *mothers’ concerns bear a temporal nature* as they keep on waxing and waning throughout pregnancy and early childhood depending on the stage of their caregiving journey. This prolonged state of fear and anxiety stems from lack of assurance about when the child can be declared as HIV negative. Our own synthesis of previous research shows that all mothers living with HIV go through a transitional state of uncertainty after childbirth, when HIV exposure continues but HIV negative diagnosis has not been established (Pandya et al., 2025). The current study further adds that irrespective of when the mother receives the last HIV negative test result of her child, in her mind, there is no fixed cut off for the duration of this transitional period. For some mothers, this period ends when breastfeeding stops or after 2 to 3 negative tests whereas for others, this period of uncertainty continues longer as their concerns reemerge during a sickness or weight loss episode in the child. Hence, we conclude that every mother living with HIV must go through a different battle. The point when she is finally relieved about her child’s HIV negative status depends on several factors as highlighted in this study including breastfeeding decisions, timing of repeated HIV tests, perceptions about ART and finally child’s overall health, growth and developmental patterns manifested during early childhood.

The factor that underpinned all mothers’ concerns was a *lack of knowledge about the process of HIV testing for their children*. Unlike diagnostic tests for other chronic diseases, a definitive diagnosis of HIV for their child is not available to mothers since it happens over a period of time and involves multiple tests, thus resulting in a liminal state full of hope, fear and uncertainty without immediate clarity for mothers (Mazanderani & Sherman, 2019; Sacks et al., 2017). It is already known that chronic intermittent uncertainty about infant’s HIV status during the period of infant testing can be detrimental to a mother’s psychological and physiological health and can affect mother-infant relationship (Karugaba et al., 2022; Shannon, 2015). Our findings further reinforce that even though HIV negative test results bring relief to mothers, the long process of testing significantly exacerbates their anxiety and concerns. It is also worth highlighting that a lot of mothers’ suspicions about ‘*HIV coming back*’ stemmed from their unawareness about modes of HIV transmission to the child after birth. In this regard, counseling services as part of current vertical HIV transmission prevention interventions need to address this gap by providing appropriate information about HIV testing process (including frequency of tests, rationale behind multiple tests and timing of final test) as well as modes of HIV transmission, early enough during pregnancy or post-partum to manage expectations and reduce anxiety.

Furthermore, we found that irrespective of the age of final negative test (conducted 6 weeks after cessation of breastfeeding or around 18 months, whichever comes later), if their child had *repeated sickness episodes, hospitalization or growth faltering issues*, mothers’ fear and anxiety reemerged. It is worth noting that in the absence of convincing explanation from health workers, while most of these mothers kept speculating about reasons for their child’s condition, many of them suspected HIV infection coming back. In terms of reasons, while biological, environmental, or socio-economic factors could have contributed to these issues, it could also be the effect of in-utero exposure to HIV and ART. It is already known from evidence that children who are HEU remain at risk and are vulnerable to suffer from higher morbidity even after ruling out HIV infection (Brennan et al., 2016; 2019; Desmonde et al., 2016; Evans et al., 2016; Sudfeld et al., 2016; Sugandhi et al., 2013; Von Mollendorf et al., 2015; Wedderburn et al., 2022). For a mother, who is already worried about her child’s overall health and future in the context of HIV exposure, dealing with any deviation in expected health, growth or development patterns in the child could be overwhelming and add a lot of distress to her preexisting fears.

Given that mothers perceive the ‘*consequences*’ or ‘*effects*’ of HIV for their child in binary terms as having or not having HIV, this study emphasizes that they remain completely unaware about any potential adverse effects of in-utero HIV or ART exposure on their child which could likely explain their child’s health, growth and development patterns during early childhood, beyond a negative test. While emerging research on effects of HIV exposure on uninfected children (including CHERISH) can throw light on the duration, severity and contribution of these effects, from a caregiving perspective, it is critical that mothers are reassured about what to expect regarding the health, growth and development of their children who are HEU in the medium to long term (once other causes of morbidity have been ruled out) (Evans et al., 2016; Prendergast & Evans, 2023; Ramokolo et al., 2019). It might be argued that excess information provided to mothers could scare them and exacerbate their preexisting concerns about long term well-being of their children who are HEU. However, it is also important to note that in the absence of any informational support, currently mothers are speculating the worst possible outcome (i.e. child having HIV) during these sickness or growth faltering episodes which significantly adds to their concerns and prolongs the period of their distress. From a policy perspective, offering the right amount of evidence based information at the right stage of motherhood, tailored to individual circumstances, risk factors and needs of mothers, can possibly reduce mothers’ persistent stress as they would not only know better about what to expect but would also be more likely to adhere to the additional health care interventions prescribed for children who are HEU such as additional monitoring and follow up of those children with risk factors - low birth weight, small for gestational age, repeated sickness, and hospitalization, poor growth, and developmental delays) (Slogrove & Cotton, 2016; Wessels et al., 2020). Furthermore, we also need to decide at a policy and program level, about the optimal information to be given to health workers about these effects since they are the first point of contact for mothers and mothers do trust them with advice.

Last but not the least, this study found that despite being relieved after a negative birth PCR, having to decide about their *child’s feeding not only triggered mothers’ concerns, but also had a major bearing on their feeding decisions, practices and experiences*, especially during the period of early infancy. Even though, a lot of mothers in our study initiated exclusive breastfeeding following advice from health workers, their doubts and fear about HIV transmission through breastmilk surpassed their knowledge about benefits of exclusive breastfeeding, resulting in early discontinuation or mixed feeding. In this regard, other factors such as health workers’ personal biases, inadequate counseling skills and guideline knowledge, cultural beliefs about feeding, stigma related to HIV and disclosure, maternal lack of decision-making power and poor support from partner and family have been shown to affect exclusive breastfeeding in mothers living with HIV (Al-Mujtaba et al., 2016; Nyoni et al., 2019; Samburu et al., 2021).

Our findings imply that the current infant feeding counseling messages received by mothers do result in successful initiation of exclusive breastfeeding as mothers get motivated and temporarily overcome their fear of HIV transmission. However, it is the continuation where gaps exist. We noted that despite the successful initiation, these mothers remained in a constant state of discomfort and anxiety which made their breastfeeding experience unpleasant. Consequently, when they faced any feeding related challenges (nipple issues, job commitments, occasional non-adherence to antiretrovirals), their concerns resurfaced, overriding their initial motivation, thus leading to premature cessation of breastfeeding. It is also worth highlighting that none of these mothers received any help or support to resume breastfeeding, after these challenges were resolved. From a supply side, this calls for a need to put more efforts to ensure continuation of breastfeeding (between 1 and 5 months) with a specific focus on those mothers living with HIV, who face feeding issues during early infancy, since they are more vulnerable and likely to discontinue. This support not only needs to address mothers’ underlying concerns about HIV transmission through breastmilk but also needs to rebuild their trust in ART and consider alternates such as expressed breast milk (especially for those with nipple issues or job commitments) (Marinda et al., 2017; Nieuwoudt et al., 2019; Omonaiye et al., 2018; Samburu et al., 2021; World Health Organization & United Nations Children’s fund, 2016).

Our study had *limitations*. Firstly, small sample size and qualitative design limit the generalizability of the findings. However, even though the results reflect perspectives from a small group of mothers in rural South Africa, we believe that our findings hold relevance for most of the mothers living with HIV across low- and middle-income countries with high antenatal prevalence of HIV. Secondly, since data was collected retrospectively, there is a chance of recall bias. Moreover, since the participants recruited were already a part of a larger prospective cohort study, in which they had multiple contacts with the research team, it is likely that these mothers might have more knowledge and awareness about health care in the context of HIV as compared to the general population. Lastly, due to the narrow focus of this study on HIV related concerns, role of structural, psychosocial and environmental factors that could have co-existed and contributed to mothers’ preexisting concerns about HIV for their children (such as their own health issues, partner and family relations, financial stress) as well as mitigating factors such as support system, were not explored in this study and warrant future research (Ashaba et al., 2017; Shannon et al., 2008).

From a caregiving perspective, *upcoming research studies* on children who are HEU, need to throw more light on relevant messages to be given to mothers including expected effects of in-utero HIV or antiretroviral exposure especially in terms of age of occurrence, how long do they last, severity and impact on children’s long-term health and well-being. Moreover, long-term follow-up studies involving caregivers/parents of children who are HEU also need to explore caregiving experiences and broader concerns of this growing population (both HIV related and unrelated) which could potentially help in better supporting caregivers and their children.

## Conclusion

As more children who are HEU are surviving and thriving, it is critical that their mothers are not ignored and supported throughout the early years of motherhood. Going forward, existing vertical HIV transmission prevention programs in South Africa and other similar settings, need to acknowledge concerns of mothers living with HIV about their children (before and beyond ruling out HIV) and how it affects their mental and physical well-being, throughout their journey of early motherhood. In this regard, scheduled contacts with health workers in facility and community as part of routine maternal-child health and HIV services, could offer a golden opportunity to discuss mothers’ concerns and provide clarifications and quick responses to their questions about HIV testing process, modes of HIV transmission, feeding dilemmas, side-effects of ART and health, growth and development of their children who are HEU.

## Figures and Tables

**Table 1.** Socio-demographic characteristics of participants.

Participant’s characteristics	Frequency (N = 20)	Percentage
Age -		
Range	22–41	
Mean (SD)	33 years (5.3)	
Cohabitation status (with partner) -		
Cohabitating	9	45
Not cohabitating	11	55
Education -		
No schooling	1	20
Junior high school (upto grade 9)	13	65
Senior High school (grade 10 to 12)	6	30
College/University	0	0
Employment status -		
Employed	4	20
Unemployed	15	75
Studying	1	5
Language spoken at home -		
English	3	15
IsiXhosa	17	85
Number of pregnancies-		
One	7	35
More than one	13	65
Number of living children-		
Mean (SD)	2.7 (1.05)	
Age of child (HIV exposed and uninfected) when enrolled in this study-		
Range	12.05–24 months	
Mean (SD)	16.7 months (3.1)	

## Appendix 1 Interview Guide

**Table:**

How was your experience of being pregnant with HIV? (describe your feelings and concerns)
How do you think your HIV affected your baby before he/she was born (while in the stomach/womb)?
Describe your thoughts, feelings, and concerns regarding HIV for the child. (from birth until now)
Describe your experiences, thoughts, and concerns about feeding your child after birth? (any concerns, challenges)
Elaborate your experience with HIV testing for the child since birth until now.
Even though your baby has been tested negative for HIV, is there anything that you wonder about, feel concerned about, or think about? (health, nutrition, growth, development etc)
What are your thoughts about your child’s overall health and well-being? What precautions/measures have you taken to ensure that your child is HIV free?
